# P20 Addition of a pharmacy technician to the ward team improves antimicrobial stewardship and patient care and safety

**DOI:** 10.1093/jacamr/dlad066.024

**Published:** 2023-06-14

**Authors:** R Rodger, Jonathan Coulter, Sharon Thompson

**Affiliations:** Royal Alexandra Hospital, NHS Greater Glasgow & Clyde; Royal Alexandra Hospital, NHS Greater Glasgow & Clyde; Royal Alexandra Hospital, NHS Greater Glasgow & Clyde

## Abstract

**Background:**

Antimicrobial stewardship (AS) is essential in reducing the risk of antimicrobial resistance (AMR).^1,2^ Prospective feedback of AS issues and adopting a multidisciplinary approach are beneficial to patient care and safety.^3^ Within the multidisciplinary team, antimicrobial pharmacy technician (AMPT) roles are evolving and offer an excellent opportunity to support everyday AS.^4^ Documenting antibiotic stop dates and allergy status on the prescribing chart and restricting broad spectrum antimicrobials to ‘protected’ status can improve AS by ensuring patients receive the correct antibiotic for the appropriate duration.^5^ In addition, management of common antibiotic drug interactions with cation products such as oral iron and enteral feeds is important in preventing treatment failure and AMR.^6^

**Objectives:**

This study set out to determine if introduction of an AMPT to the multidisciplinary team can improve AS, patient care and safety and reduce AMR risk.

**Methods:**

A quality improvement approach was used to introduce an AMPT ward service to the Royal Alexandra Hospital (RAH) over a period of 6 months. The service was developed, standardized, piloted and agreed. The AMPT visited medical, surgical and older people service wards daily and screened prescribing charts for the following: (i) documented allergy status, (ii) documented antibiotic stop/review dates, (iii) completion of ‘protected’ antimicrobial order forms, and (iv) management of antibiotic cation interactions. AS issues identified were shared prospectively with the ward team and data displayed on run charts to measure improvement.

**Results:**

Following introduction of the AMPT service documentation of allergy status and oral antibiotic stop dates improved from baseline in all wards. IV antibiotic stop/review documentation increased in all downstream medical and surgical wards with the greatest improvement achieved in the respiratory ward, increasing from a 0% baseline to a median of 37% after service introduction. Awareness and management of antibiotic cation interactions increased from a 41% baseline to a median of 86% and total antibiotic doses compromised by this interaction reduced from 66% at baseline to 21% post change. Completion of ‘protected’ antimicrobial forms improved from a baseline median of 53% to a median of 85% after service introduction.

**Conclusions:**

Introduction of an AMPT service resulted in improved documentation of allergy status, oral and IV antibiotic stop/review dates, recognition and management of antibiotic cation interactions and compliance with the local ‘protected’ antimicrobial policy. These results demonstrate that introduction of an AMPT to the ward team can improve AS, patient care and safety and reduce the risk of AMR.
